# Decreased Postural Complexity in Overweight to Obese Children and Adolescents: A Cross-Sectional Study

**DOI:** 10.3389/fnhum.2022.850548

**Published:** 2022-04-28

**Authors:** Hans-Peter Wiesinger, Michael Buchecker, Erich Müller, Thomas Stöggl, Jürgen Birklbauer

**Affiliations:** ^1^Department of Sport and Exercise Science, University of Salzburg, Salzburg, Austria; ^2^Red Bull Athlete Performance Center, Salzburg, Austria

**Keywords:** inertial measurement unit, root mean square, sample entropy, largest Lyapunov exponent, detrended fluctuation analysis, reproducibility

## Abstract

**Introduction:**

Although a few studies suggest that young overweight to obese children and adolescents (YO) may have impaired postural control compared to young normal-weight (YN) peers, little information exists about how these two groups differ in the quality of the underlying balance strategies employed. Hence, the aim of the present study was a first comprehensive examination of the structural complexity of postural sways in these two cohorts during quiet bilateral standing.

**Methods:**

Nineteen YO secondary school students (13.0 ± 1.4 years; male = 10, female = 9) were carefully matched to YN controls (13.0 ± 1.5 years) for age, sex, height, and school. Mediolateral (ML) and anteriorposterior (AP) acceleration signals were recorded with an inertial measurement unit (IMU) positioned at the trunk while standing barefoot in two conditions: firm and foam support surface. The magnitude of postural fluctuations was obtained using the root mean square (RMS). The temporal structure of the signals was analyzed *via* sample entropy (SEn), largest Lyapunov exponent (LyE), and detrended fluctuation analysis (α-DFA) algorithm. Reliability was assessed using a test–retest design.

**Results:**

In both groups, foam standing caused higher postural fluctuations (higher RMS values) and reduced structural complexity (lower SEn values, higher LyE values, higher α-DFA values). In comparison to YN, YO exhibited a higher RMS_AP_. Especially in ML direction, the acceleration signals of the YO had higher repeatability (smaller SEn values), greater long-range correlations (higher α-DFA values), and lower local stability (higher LyE values). However, these observations were largely independent of the task difficulty. Except for α-DFA_AP_, the IMU approach proved reliable to characterize posture control.

**Discussion:**

Our outcomes confirm postural control deficits in YO compared to their YN peers and indicate impaired regulatory mechanisms reflected as rigidity. Such less complex patterns usually reflect diverse pathologies, are detrimental to compensate for internal or external perturbations, and are attributed to lower adaptability and task performance. Without targeted balance stimuli, YO likely end in a lifelong vicious circle of mutually dependent poor balance regulation and low physical activity.

## Introduction

According to the most recent report published by the Non-Communicable Disease Risk Factor Collaboration, more than 340 million children and adolescents aged 5–19 years worldwide were overweight or obese (girls: 18% and boys: 19%) in 2016 (Abarca-Gómez et al., 2017). Increasing physical activity would be a valuable instrument to counteract this pediatric condition and its detrimental effects on health (Kelley et al., 2014). However, several studies have discovered a negative association between adiposity levels and physical activity participation in childhood and adolescence (Jimenez-Pavon et al., 2010; Prentice-Dunn and Prentice-Dunn, 2012). Following, for example, McGraw et al. (2000), a contributing factor for the poor adherence to physical activity in overweight to obese children and adolescents (YO) could be their well-established difficulties in maintaining balance (Steinberg et al., 2018). Consequently, YO likely feel less confident to execute basic motor tasks and thus less motivated to engage in sports and exercise than their young normal-weight peers (YN; McGraw et al., 2000; Jimenez-Pavon et al., 2010; Prentice-Dunn and Prentice-Dunn, 2012).

Postural control deficits in YO have been systematically demonstrated with static post-urography, particularly under challenging conditions like standing under proprioceptive manipulation (e.g., D’Hondt et al., 2011; Steinberg et al., 2018). Center of pressure (CoP) fluctuations, usually registered with force plates, are acknowledged as not purely random but to have a deterministic origin containing crucial information about the time-evolving dynamics of balance control. However, these structural features embedded in the time series remained ignored by previous studies, that only focused on the outcome of performance by analyzing the magnitude instead of the structure of CoP variability in YO. Best to our knowledge, only three studies examined CoP trajectories of YO at the underlying control level. They indicated proprioceptive impairments (Fink et al., 2019), less sensitivity to correct for small shifts in the CoP (Villarrasa-Sapina et al., 2016), and altered behavioral strategies governing standing (Pau et al., 2012). Given the few original and explorative studies on measures of the structure of variability, it remains unclear to what extent an excessive body mass in childhood and adolescence affects the collective regulation of the postural system, or in other words, its fundamental patterns of coordination during postural balance tasks. Hence, additional research is required to provide more detailed insights into the complexity of these regulation processes unveiled by non-linear dynamical analyses and give trainers and clinicians better knowledge to promote more sustainable exercise behavior in this specific cohort.

Unlike the perturbed conditions in previous non-linear analyses of YO, we used a foam surface to enhance the task demands in a manner that challenges the whole sensorimotor balance control system and is significant for a plethora of physical activities. In addition, we aimed to perform an exhaustive but routinely applicable assessment of postural balance in YO and YN by unfolding postural control dynamical properties *via* the first parallel use of the most prominent non-linear tools, known as sample entropy (SEn), largest Lyapunov exponent (LyE), and detrended fluctuation analysis (α-DFA; Stergiou and Decker, 2011; Mukherjee and Yentes, 2018). In brief, SEn estimates the rate of regularity, LyE extracts the trajectory divergence, and α-DFA quantifies long-range correlations (Peng et al., 1995; Richman and Moorman, 2000) in the signal in question. In contrast to the amount of variability, each of these techniques evaluates the structural characteristics embedded in the CoP trajectory from different conceptual perspectives (e.g., Goldberger et al., 2002; Stergiou and Decker, 2011). However, all output measures of SEn, LyE, and α-DFA belong to the same construct, usually called complexity (Roerdink et al., 2006; Lamoth et al., 2009; Lamoth and van Heuvelen, 2012; Mukherjee and Yentes, 2018). High complexity in human motion is thought to reflect a rich repertoire of coordinative solutions (Harrison and Stergiou, 2015) that allow the controller to readily respond to the diverse internal and external stressors encountered in daily life. Inversely, pathology, aging, increased task demands, or a lack of physical experience are prominent causes that render systems physiologically less complex and thus less adaptive (Goldberger et al., 2002; Roerdink et al., 2006; Lamoth et al., 2009; Stergiou and Decker, 2011; Lamoth and van Heuvelen, 2012; van Emmerik et al., 2016). Although not uncontroversial, a loss of complexity in CoP displacement of static posturography tests would be accompanied by a breakdown in the number of active degrees of freedom and, as a result, maladaptive responses to perturbation (Lipsitz, 2002). Accordingly, postural sway dynamics of YO and elderly typically show more repetitive (analyzed via SEn) or less locally stable (analyzed via LyE) patterns or a decrease in the fractal organization of the scaling exponent α (analyzed via α-DFA) (Ko and Newell, 2016; Villarrasa-Sapina et al., 2016).

Since single non-linear metrics cannot comprehensively quantify the complexity of a one-dimensional time series (e.g., Goldberger et al., 2002; Stergiou and Decker, 2011), the application of three complementary mathematical tools is a strength of our study. In this way, we reveal the overall impression of the temporal organization of the sway signal while reducing the risk of misinterpretations. Considering the earlier findings described above, we expected a reduced postural complexity in terms of higher repeatability (lower SEn values), lower local stability (higher LyE values), and higher long-range correlation (higher α-DFA values) in YO compared to their YN peers. Further, we hypothesized that this functional deficit would become even more evident in the foam standing condition. Since sway data were captured using simple accelerometry within a non-laboratory setting, we also tested the reliability of the methodology employed.

## Materials and Methods

A two-group cross-sectional comparative design was used to examine the hypothesis. Participating children were recruited from three local secondary schools, and all subjects were informed about the objectives, risks, and benefits of the study before providing their written consent. Signed parental permission was sought for children younger than 14 years. The project was approved by the local ethics review board (EK-GZ: 17/2018) and was conducted in accordance with the Declaration of Helsinki.

### Participants

**FIGURE 1:**
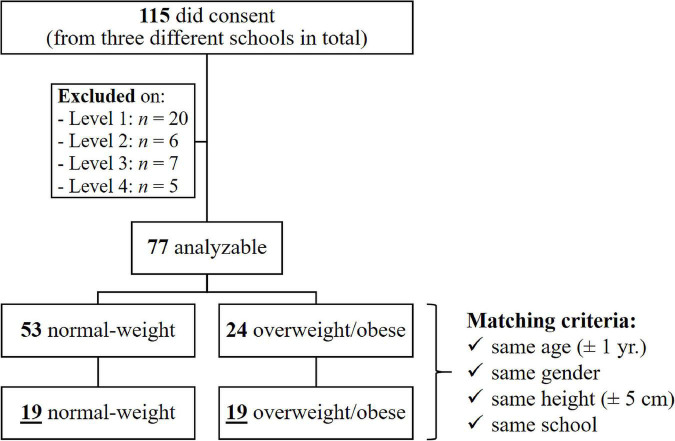
Summary of volunteer recruitment and participation. Children were excluded due to self- or parental-reported acute injury or orthopedic, neurological, cardiac, or cognitive disorders, including medication that could affect balance (Level 1), or in case one of the following applied during any test session: unavailability (Level 2), feeling unwell or non-compliant behavior (both Level 3), or corrupted signals (Level 4).

The detailed participant selection process and the participants’ characteristics are presented in Figure 1 and Table 1. In sum, 38 out of the 115 10–16-year-old YO and YN had to be excluded for further analysis due to medical, personal, or technical reasons. Of the remaining 77 children, 24 were overweight or obese, while 53 were normal-weight. In the end, 19 (overweight, *n* = 9; obese, *n* = 10) out of 24 of the YO could be *a posteriori* strictly age-, sex-, height-, and school-matched to a YN control, thus leaving 38 children to be thoroughly evaluated. Age- and sex-specific body mass index percentiles for classifying weight status among children were computed using the online calculator provided by the Centers for Disease Control and Prevention^1^ : normal-weight, 5th ≤ BMI < 85th percentile; overweight, 85th ≤ BMI < 95th percentile; obese, BMI ≥ 95th percentile). No child stated to have ever been involved in a weight loss or obesity treatment program.

**TABLE 1:** Group statistics of the young overweight/obese (*n* = 19) and their matched normal-weight controls (*n* = 19).

	Overweight/Obese	Normal-weight	*t*_36_	Difference BCa 95% CI	*p*	*f*
Girls/Boys	9/10	9/10				
Age (Years)	13.0 ± 1.4	13.0 ± 1.5	0.09	[−0.88, 0.88]	0.930	0.01
Body height (cm)	158.2 ± 7.9	157.8 ± 8.4	0.14	[−4.80, 5.15]	0.892	0.02
Body mass (kg)	64.0 ± 10.8	46.4 ± 9.6	5.32	[10.92, 24.12]	<0.001	0.89
BMI (kg⋅m^–2^)	25.4 ± 2.5	18.4 ± 2.1	9.22	[5.64, 8.44]	<0.001	1.54
BMI percentile (%)	93.4 ± 3.7	45.1 ± 22.5	9.22	[38.49, 57.35]	<0.001	1.54

*Descriptive statistics are expressed as mean ± standard deviation values. t-values, p-values, and effect size f are presented for the independent t-test. Bias-corrected and accelerated (BCa) 95 percent bootstrap confidence interval for mean differences. Age, body height, and body mass information were obtained during the first visit. BMI, body mass index.*

### Data Collection

For reliability testing, the same principal investigator accomplished experimental data collection twice within approximately 12 days (SD: ± 2 days) at the same location and at a comparable time of the day.

Children were tested individually in a quiet room at their regular school during morning classes. After height and weight assessment, the young participants were asked to stand (two-legged) as still as possible with eyes open, first, on a firm, and then on a foam (Balance-pad Solid, Airex, Switzerland) support surface. We refrained from using randomization since we expected higher transfer effects from foam to the firm condition than *vice versa*. Both trials lasted 60 s with a 2 min rest period in between. All tests were performed with minimal clothing and without shoes. The detailed instructions included (a) to look straight ahead at an eye-level target (positioned 3 m away), (b) to keep the feet hip-width apart and the arms relaxed at the sides, and (c) to breath as usual. Simultaneously, postural sway acceleration (ACC) signals were captured through a commercially available 50 mm × 70 mm × 20 mm (mass: 35 g) inertial measurement unit (IMU) (GyKo, Microgate, Italy) sampling data routinely at 500 Hz. The IMU was attached following the manufacturer’s recommendation to the children’s thoracic spine using the associated semi-elastic vest. The vest was equipped with a specific bib with magnetic support to avoid relative mediolateral (ML) and anteriorposterior (AP) IMU movements during the balance tests. The position of the IMU was noted thoroughly for reproducibility measures. All records were transferred *via* Bluetooth to a personal computer and stored as text files by the manufacturer’s RePower software version 1.1.1.7 (Microgate).

### Data Pre-processing

Raw ACC signals were initially down-sampled to 100 Hz and cropped to the middle 50 s, yielding 5,000 data points entering in-depth analysis. Further pre-processing steps involved (a) tilt-correction to obtain values concerning the anatomic frame of reference (Moe-Nilssen, 1998) and (b) bandpass filtering (Butterworth filter, 4th order, zero-lag, bandpass frequency: 0.3–10 Hz) to reduce both biases caused by respiration and by low amplitude measurement noise (Ruhe et al., 2010; Martinez-Mendez et al., 2012). Subsequently, given time series were inspected for potential (previously overlooked) abnormalities in task performance (Mancini et al., 2012). More precisely, the resultant ACC vector time series (the square root of the sum of squares of the ACC vector in ML and AP direction) was divided into five 10 s windows. The standard deviation was evaluated in each window. If one of these standard deviation values exceeded the fivefold of the smallest standard deviation value, all data from the particular child were ignored (Mancini et al., 2012). For this reason, three children had to be excluded for further analysis and another two because of unexpected signal gaps (see Level 4 in Figure 1).

### Outcome Variables Overview

The primary outcome was the measure of postural complexity, including the structure related scaling exponent α-DFA, as well as SEn and LyE estimates. The secondary outcome was the root mean square (RMS) quantifying the magnitude of sway variability. Differences in the dependent variables were assessed separately for ACC_ML_ and ACC_AP_ profiles.

### Sample Entropy

The SEn procedure indexes the predictability (regularity or orderliness) of a signal and is mathematically defined as the negative natural logarithm of the conditional probability that a time series of length *N*, having repeated itself for *M* samples within a tolerance *r*, will also repeat itself for *M* + 1 samples, but without allowing self-matches (Richman and Moorman, 2000). Thus, a SEn value becomes zero for a perfectly repeatable signal where sub-sequences exhibit the same configuration upon comparison, and *vice versa*, a perfectly random signal elicits a SEn value converging toward infinity. Healthy human functioning is located in the intermediate region of this continuum (Stergiou and Decker, 2011). Although not undisputed, pathological states typically involve more regular and constrained movements (Goldberger et al., 2002; Stergiou and Decker, 2011; van Emmerik et al., 2016). Here, the input parameters *M* and *r* were set in line with guidelines suggested by Ramdani et al. (2009), showing that the referenced estimate of the relative error of the determined SEn values across the present records was as small as possible when *M* = 3 with *r* = 0.07⋅standard deviation regarding ACC_ML_ data and *r* = 0.06⋅standard deviation regarding ACC_AP_ data.

### Largest Lyapunov Exponent

To extract LyE estimates from one-dimensional observations, an *m*-dimensional state space was reconstructed in the first step with the method of embedding delays (Rosenstein et al., 1993; Stergiou et al., 2004). Therefore, classical concepts of “average mutual information” and of “false nearest neighbors” were applied to obtain the required delay τ and the embedding dimension *m*, respectively (Stergiou et al., 2004). In fact, τ was chosen as the first minimum in the average mutual information function, and *m* when the percentage of false nearest neighbors as a function of the embedding dimension dropped to near zero. Altogether, *m* = 4 with τ = 17 samples for ACC_ML_ and with τ = 20 samples for ACC_AP_ data were found acceptable for unfolding certain attractors. In the second step, the average exponential rate of divergence of initially nearby trajectories in each reconstructed state space was calculated following Rosenstein et al. (1993). In the third and last step, LyE values were quantified as the slope of the first linear growing part (0–75 samples) of the resulting divergence curves. Finally, they were converted to bits per second by multiplying them by the sampling frequency (100 Hz). As a general rule, the larger a LyE estimate, the greater the system’s sensitivity to infinitesimal perturbations, or in other words, the lower the local dynamic stability (Roerdink et al., 2006; Donker et al., 2007; Lamoth et al., 2009).

### Detrended Fluctuation Analysis

Detrended fluctuation analysis is a modification of the random walk analysis and has been proven suitable for revealing the extent of long-range correlations in biological signals (Peng et al., 1995; Goldberger et al., 2002; Lipsitz, 2002; Harrison and Stergiou, 2015). In brief, DFA fits a power law to the time series’ average detrended fluctuations, *F*(*n*), across different box sizes (or scales), *n*, and evaluates the scaling exponent α by determining the slope of the linear regression line of the log-log graph of *F*(*n*) vs. *n* (Damouras et al., 2010). For example, if data are completely uncorrelated (white noise), the scaling exponent α = 0.5, while for the opposite extreme (brown noise) of α = 1.5, a value at any given moment is strongly correlated to the previous one. In contrast, α = 1 reflects 1/*f* (pink) noise and implies that an event at every point approximately depends equally on those from the recent and those from the very distant past (Peng et al., 1995). Referring to Peng et al. (1995) and Lipsitz (2002), this class of fractal-like (self-similar) processes can be supposed to be quite adaptive. It should be interpreted as a broad compromise between fairly “rough” and fairly “smooth” assembled control mechanisms. Of note, to more specifically address the proprioceptive feedback loop, only α values derived from a range of scales associated with the higher frequencies in postural balance signals, that is, between 2 and 10 Hz, were currently examined. Detailed theoretical and technical aspects of this approach are described in Gilfriche et al. (2018).

### Code Availability and Statistical Analyses

The SEn and LyE algorithms were taken from PhysioNet (Goldberger et al., 2000), whereas for DFA the source code provided by Gilfriche et al. (2018) was employed. These tools were implemented in Matlab software version R2020b (The MathWorks Inc., Natick, MA, United States). Data analysis, including state space reconstruction through its integrated “Predictive Maintenance Toolbox” was entirely managed with Matlab–unless otherwise specified.

For statistical analyses, data sets were initially transformed by the natural logarithm (after adding a constant of 1) to better approximate normality (Osborne, 2002). A three-way mixed analysis of variance (ANOVA) with “condition” (firm vs. foam) and “visit” (first vs. second) as the within-subject factors and “group” (YO vs. YN) as the between-subjects factor was completed for each dependent variable. In case of significant main or interaction effects, follow-up pairwise comparisons were performed using the Benjamini-Hochberg procedure (false discovery rate) to control for family wise type I error. However, if the effect of “visit” was statistically negligible, mean values were pooled across this factor before *post-hoc* testing. The Alpha level for rejecting the null hypothesis was set at 0.05. In addition, the differences between the values obtained were evaluated by computing Cohen’s (1988) effect size *f* (small: *f* > 0.10, medium: *f* > 0.25, large: *f* > 0.40). For assessing test-retest reliability, a one-way random model of intra-class correlation (ICC_1,1_) was employed (cf. Mancini et al., 2012). Based on the work of Fleiss (1986), particular results were interpreted as poor when ICC_1,1_ ≤ 0.40, fair when ICC_1,1_ ≤ 0.60, good when ICC_1,1_ ≤ 0.75, and excellent when ICC_1,1_ ≤ 1.00. IBM SPSS version 27.0 (SPSS Inc., Chicago, IL, United States) software was applied for all statistical analyses.

## Results

### Reliability

Table 2 summarizes the test–retest reliability of ACC_ML_ and ACC_AP_ measures for the YO and their matched YN controls while standing on firm and foam support surfaces. The reliability of the non-linear measures was fair to excellent, except for the α-DFA_AP_ variable in terms of the YN controls. Therefore, the reports on this variable remain a purely descriptive one (see the section below). Despite the occasional only fair reliability in some measures of YN controls, it seems justified to assume that the given experimental field approach investigates the distinct aspects of postural control with sufficient precision.

**TABLE 2:** Test–retest reliability, as expressed with the intraclass correlation coefficient_(1,1)_, of postural sway measures.

Variable	Direction	Overweight/Obese		Normal-weight	
		Firm	Foam	Firm	Foam
SEn (bit)	ML	0.74	0.75	0.81	0.57
	AP	0.76	0.86	0.71	0.43
LyE (bit⋅s^–1^)	ML	0.70	0.79	0.52	0.71
	AP	0.68	0.73	0.54	0.55
α-DFA	ML	0.64	0.75	0.59	0.69
	AP	0.51	0.52	0.40	0.20
RMS (mm⋅s^–2^)	ML	0.83	0.79	0.64	0.49
	AP	0.85	0.84	0.75	0.59

*SEn, sample entropy; LyE, largest Lyapunov exponent; α-DFA, scaling exponent α derived from detrended fluctuation analysis; RMS, root mean square; ML, mediolateral direction; AP, anteriorposterior direction.*

### Differences

**FIGURE 2:**
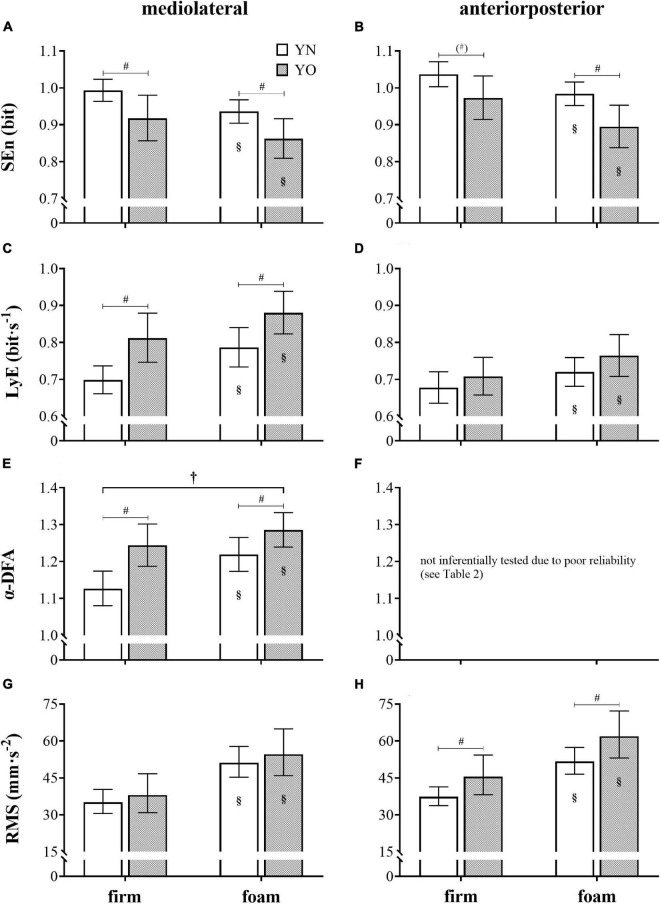
Postural complexity and magnitude of postural fluctuations. Sample entropy (SEn) **(A,B)**, largest Lyapunov exponent (LyE) **(C,D)**, scaling exponent α derived from detrended fluctuation analysis (α-DFA) **(E,F)**, and root mean square (RMS) **(G,H)** values from acceleration time series in mediolateral and anteriorposterior direction for the young overweight/obese (YO) and matched young normal-weight (YN) controls during standing on firm and foam support surfaces pooled across the visit. Bars represent anti-logged means and 95% confidence intervals. †, significant (*p* < 0.05) group × condition interaction; #, significant (*p* < 0.05) difference between groups; (^#^), nearly significant (*p* = 0.059) difference between groups; §, significantly different (*p* < 0.05) to standing on a firm support surface.

Descriptive, non-log-transformed statistics are shown in Table 3, while a summary of ANOVA results is provided in Table 4. All significant *post-hoc* comparisons are reported in Figures 2A–H.

**TABLE 3:** Descriptive statistics of postural sway measures in mediolateral and anteriorposterior direction for the young overweight/obese (*n* = 19) and their matched normal-weight controls (*n* = 19) while standing on firm and foam support surfaces according to both visits.

		Mediolateral direction				Anteriorposterior direction			
Variable	Visit	Overweight/Obese		Normal-weight		Overweight/Obese		Normal-weight	
		Firm	Foam	Firm	Foam	Firm	Foam	Firm	Foam
SEn (bit)	Visit 1	0.920 ± 0.131	0.854 ± 0.130	0.996 ± 0.065	0.933 ± 0.079	0.986 ± 0.126	0.894 ± 0.130	1.040 ± 0.068	0.982 ± 0.078
	Visit 2	0.923 ± 0.137	0.877 ± 0.096	0.992 ± 0.064	0.941 ± 0.069	0.967 ± 0.128	0.903 ± 0.114	1.036 ± 0.082	0.988 ± 0.079
LyE (bit⋅s^–1^)	Visit 1	0.817 ± 0.144	0.897 ± 0.131	0.695 ± 0.089	0.792 ± 0.123	0.702 ± 0.114	0.771 ± 0.108	0.673 ± 0.106	0.728 ± 0.105
	Visit 2	0.818 ± 0.164	0.871 ± 0.125	0.707 ± 0.094	0.789 ± 0.122	0.722 ± 0.120	0.765 ± 0.144	0.688 ± 0.099	0.717 ± 0.080
α-DFA	Visit 1	1.257 ± 0.135	1.307 ± 0.102	1.129 ± 0.112	1.215 ± 0.097	1.233 ± 0.107	1.313 ± 0.111	1.200 ± 0.108	1.239 ± 0.093
	Visit 2	1.238 ± 0.130	1.268 ± 0.103	1.129 ± 0.106	1.228 ± 0.109	1.240 ± 0.114	1.242 ± 0.157	1.208 ± 0.100	1.207 ± 0.108
RMS (mm⋅s^–2^)	Visit 1	40.3 ± 16.8	61.1 ± 30.5	34.7 ± 11.6	51.2 ± 14.5	47.4 ± 18.3	65.8 ± 26.1	37.1 ± 8.5	52.6 ± 13.4
	Visit 2	43.2 ± 24.3	56.1 ± 17.1	38.8 ± 11.4	55.4 ± 18.4	50.5 ± 24.4	64.8 ± 20.0	39.5 ± 10.1	53.9 ± 15.5

*Data are expressed as mean ± standard deviation values. SEn, sample entropy; LyE, largest Lyapunov exponent; α-DFA, scaling exponent α derived from detrended fluctuation analysis; RMS, root mean square.*

**TABLE 4:** Summary of inferential ANOVA statistics. *F*-values, *p*-values, and effect size *f* are presented for a three-way mixed analysis of variance.

Variable	Direction	Statistics	Group	Condition	Visit	Group × Condition	Group × Visit	Condition × Visit	Group × Condition × Visit
SEn (bit)	ML	*F*_1,36_	6.11	39.78	0.81	0.00	0.41	0.95	0.11
		*p*	0.018	<0.001	0.373	0.996	0.528	0.336	0.739
		*f*	0.41	1.05	0.15	0.00	0.11	0.16	0.06
	AP	*F*_1,36_	6.34	42.15	0.03	1.83	0.06	2.15	0.47
		*p*	0.016	<0.001	0.867	0.184	0.801	0.152	0.497
		*f*	0.42	1.08	0.03	0.23	0.04	0.24	0.11
LyE (bit⋅s^–1^)	ML	*F*_1,36_	9.05	40.39	0.09	0.93	0.40	1.55	0.08
		*p*	0.005	<0.001	0.765	0.341	0.532	0.221	0.779
		*f*	0.50	1.06	0.05	0.16	0.11	0.21	0.05
	AP	*F*_1,36_	1.56	16.15	0.10	0.26	0.01	2.84	0.00
		*p*	0.220	<0.001	0.749	0.613	0.914	0.100	0.945
		*f*	0.21	0.67	0.05	0.08	0.02	0.28	0.01
α-DFA	ML	*F*_1,36_	8.81	32.38	0.72	5.10	1.76	0.06	0.95
		*p*	0.005	<0.001	0.403	0.030	0.194	0.810	0.337
		*f*	0.49	0.95	0.14	0.38	0.22	0.04	0.16
	AP	*F*_1,36_							
		*p*	not inferentially tested due to poor reliability (see Table 2)						
		*f*							
RMS (mm⋅s^–2^)	ML	*F*_1,36_	0.45	197.05	2.33	0.06	1.83	1.36	0.12
		*p*	0.505	<0.001	0.136	0.806	0.185	0.251	0.734
		*f*	0.11	2.34	0.25	0.04	0.23	0.19	0.06
	AP	*F*_1,36_	4.47	169.39	1.80	0.10	0.02	1.09	0.01
		*p*	0.042	<0.001	0.188	0.748	0.878	0.303	0.942
		*f*	0.35	2.17	0.22	0.05	0.03	0.17	0.01

*SEn, sample entropy; LyE, largest Lyapunov exponent; α-DFA, scaling exponent α derived from detrended fluctuation analysis; RMS, root mean square; ML, mediolateral direction; AP, anteriorposterior direction.*

There was no significant main effect of visit nor any group × visit, condition × visit, or group × condition × visit interaction across the variables considered (all *p* > 0.05). Each outcome measure revealed a significant main effect of condition (all *p* < 0.001). More precisely, SEn_ML_ (YO: *p* < 0.001, *f* = 1.09; YN: *p* < 0.001, *f* = 1.02) and SEn_AP_ (YO: *p* < 0.001, *f* = 1.24; YN: *p* = 0.001, *f* = 0.91) values were lower for standing on a foam compared to standing on a firm support surface, whereas the LyE_ML_ (YO: *p* < 0.001, *f* = 0.97; YN: *p* < 0.001, *f* = 1.14), LyE_AP_ (YO: *p* = 0.003, *f* = 0.87; YN: *p* = 0.039, *f* = 0.52), α-DFA_ML_ (YO: *p* = 0.031, *f* = 0.55; YN: *p* < 0.001, *f* = 1.37), RMS_ML_ (YO: *p* < 0.001, *f* = 2.13; YN: *p* < 0.001, *f* = 2.61), and RMS_AP_ (YO: *p* < 0.001, *f* = 1.84; YN: *p* < 0.001, *f* = 2.70) variables exhibited higher values in the foam than in the firm condition.

A significant main effect of group was found for the SEn_ML_, SEn_AP_, LyE_ML_, α-DFA_ML_, and RMS_AP_ (all *p* < 0.05) but not for the LyE_AP_ and RMS_ML_ variables (all *p* > 0.05). In detail, the YO had smaller SEn_ML_ (firm: *p* = 0.030, *f* = 0.38; foam: *p* = 0.030, *f* = 0.41) and SEn_AP_ (firm: *p* = 0.059, *f* = 0.33; foam: *p* = 0.018, *f* = 0.47) values as well as increased LyE_ML_ (firm: *p* = 0.006, *f* = 0.53; foam: *p* = 0.017, *f* = 0.42), α-DFA_ML_ (firm: *p* = 0.004, *f* = 0.56; foam: *p* = 0.040, *f* = 0.35), and RMS_AP_ (firm: *p* = 0.049, *f* = 0.34; foam: *p* = 0.049, *f* = 0.34) values compared to their YN controls.

No group × condition interaction was detected throughout (all *p* > 0.05) except for the α-DFA_ML_ variable (*p* = 0.030), where the differences between groups were noticed to be more pronounced in the firm compared to foam condition.

## Discussion

In agreement with our primary hypothesis and the loss-of-complexity hypothesis (Lipsitz and Goldberger, 1992), YO display postural control deficits with a more conservative and constrained balance strategy in double-legged quiet standing than their YN peers. We were the first to show that this decomplexification of postural fluctuations in YO have already been clearly detectable during firm standing. We expand this finding to standing under proprioceptive manipulation and provide evidence that the regulation of the trunk becomes systematically more repeatable, less fractally organized, and more sensitive to initial perturbations on a foam surface. However, contrary to our secondary hypothesis, the sway differences between YO and YN remained largely independent of the task difficulty (Figures 2A–H). Another crucial discovery of the present study was that a single inexpensive IMU enables linear and non-linear trunk excursion analyzes of similar reproducibility to gold-standard tests on a force plate (Table 2). Subsequently, this IMU approach featured a high construct validity and proved sensitive to discriminating strategies

in the underlying organization of postural control between YO and YN of the firm and foam bilateral static stance.

At a meta-level, these main outcomes of reduced balance control caused by a lower complexity in YO are broadly in line with the few previous cross-comparison studies in this cohort (Pau et al., 2012; Villarrasa-Sapina et al., 2016; Fink et al., 2019). However, past reports did not follow a comprehensive approach to reveal the temporally evolving dynamics of postural control by employing three prominent non-linear measures. Furthermore, non-neglectable methodological and sample-based differences (e.g., the number of included data points, different data pre-processing techniques) make it difficult to contextualize our structure-based outcomes in the available literature. Still, to classify our findings within this research framework, the increased magnitudes of fluctuations in the sagittal plane when being overweight or obese are consistent findings of cross-comparison studies (McGraw et al., 2000; Deforche et al., 2009; Pau et al., 2012). Likewise, the lack of between groups RMS disparity in the frontal plane is in line with our expectation of minor task challenge due to the high base of support of the hip-width double-legged stance. However, this appearance of more significant postural control deficits in the AP direction seems to emerge reversed when looking beyond the postural performance at an outcome level. That means, SEn, LyE, and the α-DFA values systematically provided group differences with pronounced decomplexification in the ML direction. These discrepancies in the outcome of magnitude (RMS) and structure-based variables (SEn, LyE, α-DFA) highlight that postural control is embedded in a complex network composed of many interacting heterogeneous constituents and underpins the value of monitoring postural control from the perspective of complexity.

Cumulatively, the used non-linear sway measures during bilateral stance on a firm or foam support surface show less complex balance strategies for YO than YN peers. Such patterns of decomplexification of postural regulation are thought to reduce the adaptability of YO to heterogeneous stressors of the natural environment but bring them into a state of increased postural challenge or even a state comparable to persons with different pathologies. Accordingly, CoP variabilities of lower SEn (Hansen et al., 2017; Lubetzky et al., 2018), higher LyE (Lamoth et al., 2009; Lamoth and van Heuvelen, 2012; Buchecker et al., 2018), and higher α-DFA values (Zhou et al., 2013) have systematically been observed across various cohorts during balance conditions like standing on unstable surfaces, perturbing the optical flow, or desensitizing the somatosensory system. Likewise, decomplexifications in postural control indicate pathologies affecting selective neuromuscular or sensory control systems (see Goldberger, 1996 for review) or age-related deteriorations (Sturnieks et al., 2008). Concerning task difficulty, the foam condition deteriorated postural performance in both groups of the present study. However, contrary to our second hypothesis and previous non-linear observations in a comparable cohort (Pau et al., 2012; Villarrasa-Sapina et al., 2016; Fink et al., 2019), standing under altered sensory condition exhibited no amplification of detrimental balance effects in the YO compared to their YN peers (Figures 2A–H). Instead, the contrary, group differences in α-DFA_ML_ values between YO and YN were even more significant in the firm than in the foam condition (Figure 2E). We speculate that this task independence reflects that standing on a foam support surface even pushed YN to their postural equilibrium limits and impeded better group discriminations.

Nevertheless, the present findings clearly show that the balance strategy of YO is less complex than the regulation processes of their YN peers. Based on models of optimal movement (Stergiou et al., 2006) or optimal coordination (Hamill et al., 2012) variability, it is strongly indicated to foster the degree of variability–particularly in YO–up a certain level where the balance control system develops a highly complex, chaotic structure. Such a system brings multiple degrees of freedom at different spatio-temporal scales into proper relations so that it flexibly compensates and quickly adapts to internal and external perturbations. At an optimal degree of temporal structuredness (Stergiou et al., 2006) and an optimal coupling variability (Hamill et al., 2012), the control system is said to become globally more stable with an improvement in task performance. From a practical perspective, state-of-the-art training concepts, like the constraints-led approach (Davids et al., 2008) or differential learning (Schöllhorn et al., 2012), aim to encourage movement complexity by prompting self-organization processes through the exploration of different movement solutions. In a recent quantitative meta-analysis (Tassignon et al., 2021), differential learning was more effective than at least traditional training methods. The constraint-led approach can be even more effective by adding specific task constraints that further stimulate exploration of the solution space (Gray, 2020). However, finding the optimal movement complexity and the optimal dose of practice variability for a given task and a certain individual in the time course of learning is still an open question, for which the assessment of the actual level of motion (postural) complexity with a toolbox of non-linear measures provides future relief.

Using a cost- and time-effective procedure in the form of one single IMU and one single 60 s balance test for judging the temporal structure of sway patterns of balance is a strength of the present paper. Intriguingly, the RMS measures and most non-linear tools provided ICC values comparable to the gold-standard force plate CoP evaluations (see Ruhe et al., 2010 for review). Data reproducibility was further confirmed by observing no relevant changes between test sessions means of any main factorial ANOVA model (*a priori* disregarding α-DFA_AP_). It is reasonable to assume that the reliability can be further improved by using the mean sway patterns of multiple similar double-legged tests within a short break. This may also advance the reproducibility of DFA_AP_ up to an acceptable level. Besides being reliable, highly economical, and practical, IMU signals have frequently been connected to smartphone applications (e.g., Aqueveque et al., 2020). Moving forward, such applications could open new possibilities for enabling real-time feedback in self-organization routines such as Newell’s Constraints Led Approaches. Therefore, the shown applicability can be crucial for opening the door to a vast array of new exercise-based therapeutic perspectives in postural control for clinicians and other practitioners.

## Conclusion

Our findings replicated previous observations showing that YO are more disadvantaged in their coordination and balance ability than normal-weight peers. Our novel approach using a simple IMU by extending traditional magnitude-based methods through implementing the most valued non-linear tools revealed underlying control processes toward a less complex but more simplified balance regulation in this specific cohort. This increased system rigidity with decreased degrees of freedom and increased sensitivity to internal perturbations is unisonously described as a disability in motor development. Since balance/stability is an essential prerequisite of almost all movement skills, we strongly urge to implement postural challenges in the early years to avoid life-long physical inactivity and all the negative consequences.

Likewise, we hope future studies delve even more into the mechanisms underlying the harmful effects of being overweight and its reciprocal impact on physical activity. Besides studies to examine the usefulness of targeted exercises, prospective investigations are urgently required to quantify the long-term effects of improvements in postural control. Consequently, much research remains to be done, yet we are confident of the high potential of balance training to help to alleviate the global epidemic of inactivity and overall mortality.

## Data Availability Statement

The raw data supporting the conclusions of this article will be made available by the authors, without undue reservation.

## Ethics Statement

The studies involving human participants were reviewed and approved by the Ethics Review Board of the University of Salzburg (EK-GZ: 17/2018). Written informed consent to participate in this study was provided by the participants’ legal guardian/next of kin.

## Author Contributions

H-PW, MB, EM, TS, and JB: conceptualization, writing—review and editing, and supervision. H-PW, MB, and TS: methodology. MB and H-PW: investigation, data analysis, and visualization. H-PW, MB, and JB: writing – original draft. All authors read and approved the content of the final manuscript.

## Conflict of Interest

TS was employed by the company Red Bull Athlete Performance Center. The remaining authors declare that the research was conducted in the absence of any commercial or financial relationships that could be construed as a potential conflict of interest.

## Publisher’s Note

All claims expressed in this article are solely those of the authors and do not necessarily represent those of their affiliated organizations, or those of the publisher, the editors and the reviewers. Any product that may be evaluated in this article, or claim that may be made by its manufacturer, is not guaranteed or endorsed by the publisher.

## Footnotes

1
https://www.cdc.gov/healthyweight/bmi/calculator.html
